# Advancing a Vision Foundation Model for Ming-Style Furniture Image Segmentation: A New Dataset and Method

**DOI:** 10.3390/s25010096

**Published:** 2024-12-27

**Authors:** Yingtong Wan, Wanru Wang, Meng Zhang, Wei Peng, He Tang

**Affiliations:** 1 School of Industrial Design, Hubei University of Technology, Wuhan 430068, China; 2211212105@hbut.edu.cn (Y.W.); 20231065@hbut.edu.cn (M.Z.); 20181016@hbut.edu.cn (W.P.); 2 School of Software Engineering, Huazhong University of Science and Technology, Wuhan 430074, China; hetang@hust.edu.cn

**Keywords:** cultural heritage preservation, image segmentation, vision foundation model, prompt learning

## Abstract

This paper tackles the challenge of accurately segmenting images of Ming-style furniture, an important aspect of China’s cultural heritage, to aid in its preservation and analysis. Existing vision foundation models, like the segment anything model (SAM), struggle with the complex structures of Ming furniture due to the need for manual prompts and imprecise segmentation outputs. To address these limitations, we introduce two key innovations: the material attribute prompter (MAP), which automatically generates prompts based on the furniture’s material properties, and the structure refinement module (SRM), which enhances segmentation by combining high- and low-level features. Additionally, we present the MF2K dataset, which includes 2073 images annotated with pixel-level masks across eight materials and environments. Our experiments demonstrate that the proposed method significantly improves the segmentation accuracy, outperforming state-of-the-art models in terms of the mean intersection over union (mIoU). Ablation studies highlight the contributions of the MAP and SRM to both the performance and computational efficiency. This work offers a powerful automated solution for segmenting intricate furniture structures, facilitating digital preservation and in-depth analysis of Ming-style furniture.

## 1. Introduction

Ming-style furniture, a jewel of China’s cultural and artistic legacy, embodies the historical progression and cultural inheritance of the nation. Ming-style furniture is a collective term for furniture styles from the Ming Dynasty to the early Qing Dynasty, primarily made of hardwood materials. During the Ming Dynasty, with the development of agriculture, the stabilization of the political situation, the advancements in water conservancy, and the growth of commerce and industry, the economy became prosperous, which resulted in unprecedented progress in furniture design.

There were several reasons for its flourishing. The implementation of an official artisan system during the Ming Dynasty allowed craftsmen to access the government service department that relied on their woodworking skills. This not only elevated the social status of craftsmen but also greatly promoted the development of the furniture industry. Design was oriented toward solving practical needs, aiming to meet societal demands in the most rational way under specific conditions. Over 130 gardens were newly constructed in Suzhou during the Ming Dynasty, creating a strong demand for a large amount of Ming-style furniture to match them. The flourishing garden construction in Suzhou indicated a powerful social need and an optimal opportunity for the development of Ming-style furniture. The lifting of seaborne trade restrictions in the Ming Dynasty facilitated frequent exchanges with countries in Southeast Asia, the South China Sea, and Central Asia, leading to large quantities of hardwood lumber being imported, such as ebony, rosewood, huanghuali, and zitan. The application of these hardwood materials also promoted advancements in woodworking tools.

Traditional Chinese furniture drew inspiration from Song Dynasty furniture, and developed through a dual exploration of functionality and aesthetics, which evolved significantly during the Ming Dynasty. At that time, the basic functionalities of furniture had been achieved, and citizens paid more attention to the implicit needs of daily life. In order to achieve a lifestyle of elegance and leisure, scholars and the literati preferred to directly participate in furniture design. The ancient book entitled *Treatise on Superfluous Things* categorized various types of furniture and provided design standards for furniture, such as couches, desks, chairs, tables, cabinets, and beds. In addition, Ming-style furniture, also known as scholarly furniture, embodies cultural sophistication and aesthetic refinement in its forms and structures. For instance, the design of the hat office chair symbolizes career advancement, while the round-back chair represents integrity and the gentlemanly spirit. The surface treatment of the furniture typically involved a waxing process, emphasizing the natural color and grain of the wood, thereby showcasing the unique, understated elegance of the material.

Celebrated for its simplicity, elegance, and proportion, the style of Ming furniture not only reached the apogee of art within the Chinese furniture tradition but also exerted a profound influence on global furniture design. The iconic Chinese chair designed by Danish designer Hans Wegner illustrates the influence of Ming-style furniture and is a typical case that combines inspiration and innovation. Similarly, the Wabi-Sabi spatial design, spearheaded by Belgian designer Axel Vervoordt, attracted a global audience because he applied Ming-style furniture as a central and luminous component to adorn such aesthetic spaces. Recognized as one of the three major furniture styles globally, alongside Western Rococo and Gothic furniture, Ming-style furniture’s compatibility with modern interior design styles significantly impacts customer satisfaction when purchasing such furniture. This also highlights the significance of Ming-style furniture in interior decoration.

Recently, deep learning for furniture image analysis has emerged [1,2,3]. In addition, the ability to digitally segment and preserve the exquisite designs, carving patterns, and joinery techniques of Ming-style furniture allows for detailed scholarly examination without compromising the integrity of the physical artifacts, thereby facilitating the digital conservation and in-depth analysis of this cultural heritage. Image segmentation is one of the most fundamental areas of study in computer vision, playing a vital role in enabling machines to perceive and understand the visual world. Image segmentation techniques are typically categorized into three types: semantic segmentation [4,5,6], instance segmentation [7,8], and panoptic segmentation [9,10]. Semantic segmentation classifies an image at the pixel level, where each pixel is assigned to a semantic class by a prediction model [11,12,13]. It is worth noting that the segmented masks produced by a semantic segmentor ignore the boundaries between different components of the image. On the other hand, instance segmentation aims to identify and segment each object instance, where the segmented masks of objects within the same category are treated separately. Panoptic segmentation is a more comprehensive approach that combines both semantic and instance segmentation. It provides a unified solution by segmenting all the pixels in an image while distinguishing between different object instances and background classes, ensuring no pixel is left unclassified.

In this paper, the segmentation of Ming-style furniture images is defined as a one-class segmentation problem, similar to salient object segmentation [14,15]. This is because we focus solely on the regions of the Ming-style furniture foreground, and the extracted furniture regions can be further analyzed for their aesthetics and design. Recently, the large vision foundation model SAM [16,17] has demonstrated remarkable capability in segmenting arbitrary entities in the visual world. However, there are two limitations when directly segmenting an image containing Ming-style furniture using SAM: (1) at least one manual prompt is required to enhance the segmentation quality for each input image; and (2) the mask predicted by the vanilla SAM tends to be coarse, especially when dealing with the complex structure of the furniture.

To overcome the aforementioned limitations, we propose (1) a material attribute prompter (MAP) to automatically learn an optimal prompt for each input image and (2) a structure refinement module (SRM) to refine the predicted mask. The MAP consists of a set of contextual prompts and a set of basic prompts, initialized by the coarse segmentation map and a zero map, respectively. Both contextual and basic prompts are learnable and adaptable for attribute-specific prompts. The SRM incorporates both the output of the mask decoder and the original image; in this way, the semantic features are enhanced by low-level features to improve the structure of the final segmentation.

In addition, we have collected and constructed a new dataset, MF2K, to facilitate this study. The MF2K dataset is specifically designed for Ming-style furniture image segmentation and comprises 2073 images, each containing at least one piece of Ming-style furniture and pixel-level masks. Additionally, the dataset includes eight different furniture materials, distributed across eight distinct environments.

In summary, the contributions of this paper are as follows:

1. A new dataset, MF2K, is proposed as the first image segmentation dataset for Ming-style furniture, to the best of our knowledge. The dataset contains 2073 Ming-style furniture images, which cover eight different environments, and the samples from each environment are relatively balanced. In addition, we provide pixel-level annotations for each image that indicate the mask of the Ming-style furniture.
2. We introduce the material attribute prompter (MAP), which provides prompts to automatically segment objects. It integrates both basic prompt (BP) and contextual prompt (CP) mechanisms to produce material-specific prompts.
3. We also propose the structure refinement module to improve the details of segmentation. It integrates high-level semantic features from the encoder with low-level details from the original image.
4. The performance of the proposed method outperforms state-of-the-art models on the MF2K dataset in terms of both the mIoU and the tuned parameters.

## 2. Literature Review

### 2.1. Vision Foundation Models and Applications

The SAM (segment anything model) [16] is a vision foundation model for image segmentation. It was built in a data collection loop with over 1 billion masks on 11 M images. Apart from the image, the SAM allows various prompt inputs to extend the zero-shot capability for unknown classes and tasks. Several works include applications of the SAM. Ma et al. [18] extended the success of SAM to medical images by fine-tuning the model with a large-scale dataset over 200 K masks across 11 different modalities. Zhang et al. [19] explored the personalization of SAM using specific visual concepts and provided a training-free PerSAM and a fine-tuning PerSAM-F. Moreover, the SAM has also been extended to remote sensing scenes [20], where 105,090 high-resolution remote sensing images and 1,668,241 instances were used to scale up the SAM. Despite the success of the SAM and its variations, the previous works require careful prompt design before automated segmentation; hence, they cannot be directly transferred to Ming-style furniture image segmentation.

### 2.2. Salient Object Detection

Salient object detection (SOD) aims to predict the pixel-level masks of the objects that attract human visual attention. GateNet [15] designed a gated dual-branch structure to establish a cooperative relationship between features of different levels to increase the network discriminability. ICON [14] introduced three diverse feature aggregations, an integrity channel enhancement, and part–whole verification to SOD. Zhao et al. [21] analyzed whether the depth was really important at the inference stage of the RGB-D SOD. Since we define the Ming-style furniture image segmentation as a special SOD task, several SOD models [14,15,21,22,23,24,25] are adopted for comparison to verify the effectiveness of the proposed method.

## 3. The Proposed Dataset MF2K

### 3.1. Dataset Collection

The MF2K dataset was constructed specifically for the segmentation of Ming-style furniture images. Ming-style furniture, a significant part of China’s cultural heritage, is known for its intricate designs and unique craftsmanship. The images in this dataset were collected from various sources, including museums, furniture showrooms, and private collections. Each image contains at least one piece of Ming-style furniture, arranged in indoor and outdoor environments. The dataset aims to preserve the artistic and historical value of these artifacts while facilitating research in image segmentation, especially for digitally preserving and analyzing fine details such as carving patterns and joinery techniques.

### 3.2. Dataset Distribution Analysis

The MF2K dataset contains a total of 2073 images, categorized based on two major factors: furniture materials and environmental settings. As shown in Table 1, the dataset features eight distinct furniture materials: Burmese rosewood, black walnut, Siamese rosewood, yellow rosewood, chicken wing wood, Tielimu, Ceylon ebony, and Chinese rosewood. Each material is represented in eight different environments, including a dining room, tearoom, corner, living room, study, courtyard, bedroom, and hall.

The following is a breakdown of the data distribution by material and environment:

- Burmese rosewood: A total of 359 images, spread across different environments such as corners (117) and living rooms (56).
- Black walnut: A total of 326 images, with 84 from living rooms and 41 from dining rooms.
- Siamese rosewood: A total of 378 images, mainly in corners (91) and living rooms (92).
- Yellow rosewood: A total of 343 images, primarily in corners (135).
- Chicken wing wood: A total of 345 images, with a significant portion in corners (114) and living rooms (54).
- Tielimu: A total of 254 images, with 86 in corners and 34 in courtyards.
- Ceylon ebony: A total of 332 images, including 86 from corners and 74 from living rooms.
- Chinese rosewood: A total of 332 images, predominantly from corners (94).

**Figure 1:**
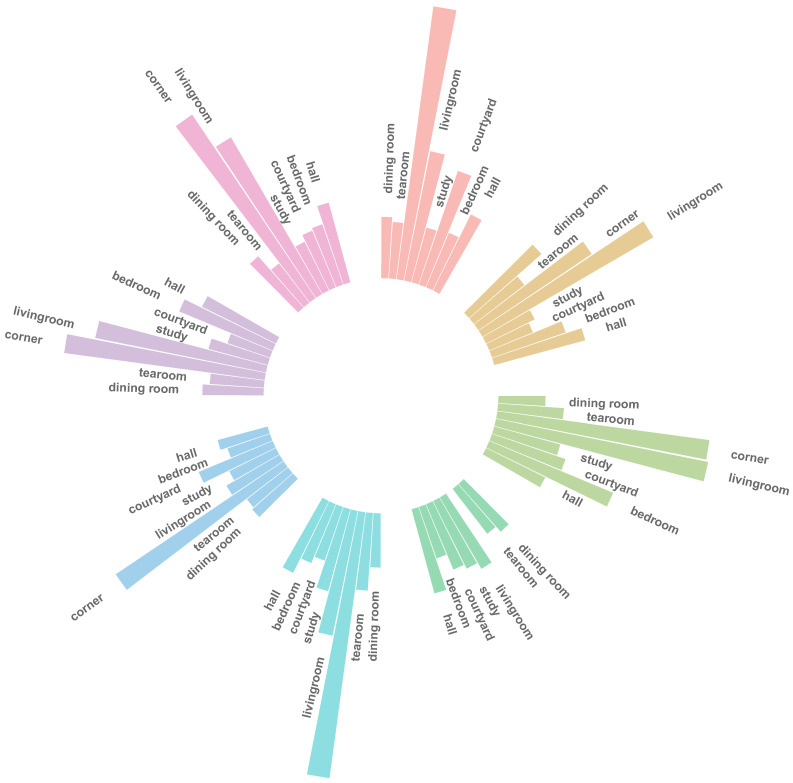
Sunburst chart of the taxonomic structure for the material and environment statistics of our dataset MF2K.

Figure 1 demonstrates how the dataset comprehensively covers diverse materials and spatial environments, ensuring that various aspects of Ming-style furniture design are captured.

### 3.3. Dataset Annotation

Each image in the MF2K dataset has been carefully annotated with pixel-level masks to identify regions containing Ming-style furniture. The segmentation process was performed to highlight the fine details of the furniture, such as carving patterns and structural elements, while ignoring background elements. A one-class semantic segmentation approach was adopted, focusing solely on the furniture regions.

To address the complexity of the segmentation task, the annotations were refined using a combination of manual labeling and automatic tools. This hybrid approach ensured the accuracy of the pixel-level masks, especially when dealing with intricate furniture structures. The annotated data allow researchers to analyze and study Ming-style furniture designs in great detail without physically handling the objects, contributing to both digital preservation and the advancement of image segmentation techniques.

## 4. Materials and Methods

In this section, we start with a brief introduction to the segment anything model (SAM) to better understand the baseline that we adopted. Then, we describe the proposed material attribute’s prompt tuning in detail, which trains the SAM in a parameter-efficient fine-tuned manner.

### 4.1. Introduction of the Segment Anything Model

The SAM [16] is an interactive prompting-enabled model architecture for category-agnostic segmentation. Specifically, the SAM consists of an image encoder, a prompt encoder, and a lightweight mask decoder. The image encoder is pretrained using the masked autoencoder (MAE) [26] with vision transformers [27]. The prompt encoder handles dense and sparse inputs such as points, boxes, and masks to solve a range of downstream segmentation problems. The mask decoder predicts the masks based on the encoded embeddings, prompt embeddings, and an output token. In summary, the full workflow of SAM can be formulated at a high level as

(1) $$M=D^{m}\left(E^{p}\left(\delta \right),E^{i}\left(I\right)\right),$$

where $E^{i}$, $E^{p}$, and $D^{m}$ denote the image encoder, prompt encoder, and mask decoder, respectively; I and M denote the input image and the output mask of the SAM, respectively; and δ denotes the manual prompt for each input I. The manual prompt δ can be either points, boxes, or masks; it varies with each input image and the human inputs.

### 4.2. SAM-Adapter Encoder

**Figure 2:**
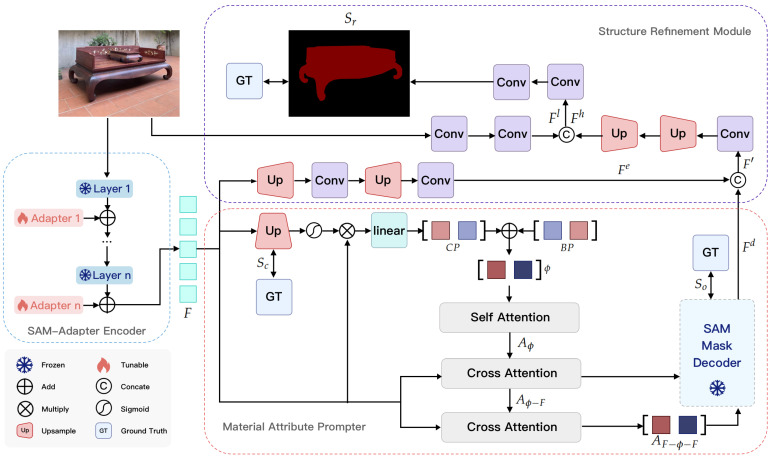
Overall architecture of the proposed network.

To make use of the SAM’s general knowledge while adapting it for downstream tasks, adapters [28] are added to the SAM’s encoder. This provides an efficient parameter-sparing method for fine-tuning the SAM. As shown in Figure 2, these adapters are placed within each encoder layer, combining task-specific insights with the broader knowledge that the main model possesses. As a result, the SAM-Adapter encoder produces an output image embedding *F* that contains features optimally suited for Ming-style furniture segmentation in the downstream task.

### 4.3. Material Attribute Prompter

According to Equation (1), the prompt δ is independent of the input image I and cannot be learned from downstream datasets. To overcome this limitation, we propose a learnable material attribute prompter (MAP) as a plug-in to the SAM. Different from Equation (1), the SAM with the proposed MAP can be formulated at a high level as Equation (2). In this approach, the learned prompt θ can be considered to be the parameters of the prompt encoder $E^{p}$. Additionally, the weights of θ are fixed once the training is completed.

(2) $$M=D^{m}\left(E_{\theta }^{p},E^{i}\left(I\right)\right).$$

As shown in Figure 2, the MAP integrates both basic prompt (BP) and contextual prompt (CP) mechanisms to produce material-specific prompts. The BP consists of learnable query embeddings that capture general attributes, and it is initialized with zeros. For the DP, the network initially extracts features *F* from the encoder, which are then used to predict a coarse map. This map undergoes processing using a sigmoid function to create an attention map $S_{c}$, which is subsequently multiplied element-wise with the original features *F* to isolate information specific to certain attributes. Further refinement is achieved by passing these features through a linear layer, resulting in the CP capturing nuanced specific attributes within each sample. The process for generating the CP is outlined in the following formula:

(3) $$\begin{array}{c} CP:=\left[Q_{F}^{C},Q_{B}^{C}\right]=Linear\left(\sigma \left(U\left(F\right)\right)⊗F\right), \end{array}$$

where $Q_{F}^{C}$ and $Q_{B}^{C}$ represent the components for CP of the foreground and background, respectively. The symbol ⊗ denotes element-wise multiplication. σ denotes the sigmoid function, and *U* is the operation to generate a coarse map by upsampling the features *F*. Linear stands for a linear function that processes the output. Unlike basic queries, which are fixed after training, the CP changes according to the image embedding *F*, making it highly adaptable and capable of explicitly capturing the discriminative material attribute of each furniture image. In this way, the CP captures the specific information, whereas the BP discerns the general differences between the foreground and background.

We sum the BP and CP to generate the complementary and adaptive prompt ϕ for the vision foundation model as

(4) $$\phi =CP+BP=\left[Q_{F}^{C},Q_{B}^{C}\right]+\left[Q_{F}^{B},Q_{B}^{B}\right]=\left[Q_{F}^{C}+Q_{F}^{B},Q_{B}^{C}+Q_{B}^{B}\right].$$

Then, we compute the self-attention of the query ϕ as

(5) $$A_{\phi }=softmax\left(\frac{Q_{\phi }K_{\phi }^{T}}{\sqrt{d}}V_{\phi }\right),$$

where $Q_{\phi }=\phi \times W^{Q}$, $K_{\phi }=\phi \times W^{K}$, and $V_{\phi }=\phi \times W^{V}$, and the $W^{Q}$, $K^{Q}$, and $K^{Q}$ are the weight matrices for the query ϕ. After that, the output of $A_{\phi }$ is considered as the query to the image embedding *F*, and we compute the cross attention with a multi-layer perceptron (MLP) as the material attribute prompt:

(6) $$A_{\phi -F}=MLP\left(softmax\left(\frac{Q_{A_{\phi }}K_{F}^{T}}{\sqrt{d}}V_{F}\right)\right),$$

where $Q_{A_{\phi }}=A_{\phi }\times W^{Q}$, $K_{F}=F\times W^{K}$, and $V_{F}=F\times W^{V}$, and the $W^{Q}$, $K^{Q}$, and $K^{Q}$ are the learnable weight matrices. In addition, the image embedding *F* is considered as the query to the material attribute prompt, and we compute the cross attention with an MLP as

(7) $$A_{F-\phi -F}=MLP\left(softmax\left(\frac{Q_{F}K_{\phi -F}^{T}}{\sqrt{d}}V_{\phi -F}\right)\right).$$

The output embedding $A_{F-\phi -F}$ is directly fed into the SAM mask decoder to provide the refined image feature.

### 4.4. Structure Refinement Module

Due to the downsampling operation in the encoder layer, the produced segmentation map often suffers from a loss of spatial resolution and fine-grained details, which is particularly problematic when segmenting intricate and complex objects such as Ming-style furniture. The coarse segmentation map may fail to capture the detailed edges, fine carving patterns, and delicate structures of the furniture, resulting in a lack of precision in the predicted mask. To address this, we propose a structure refinement module (SRM), which integrates high-level semantic features from the encoder with low-level details from the original image, allowing for a more accurate and refined segmentation output. By enhancing the structural information at multiple levels, the SRM ensures that the segmentation maps better capture the complexity and intricacy of the furniture, leading to more precise and aesthetically accurate results.

As shown in Figure 2, the image embedding *F* is directly upsampled and convolved twice to produce the feature $F^{e}$ and then concatenated with the output embedding $F^{d}$ as

(8) $$F^{\prime }=Cat\left(F^{e},F^{d}\right),$$

where $F^{e}=Conv\left(U\left(Conv\left(U\left(F\right)\right)\right)\right)$, the concatenated feature $F^{\prime }$ is convolved and upsampled twice to produce the high-level feature $F^{h}$, and it meets the dimension of the low-level feature $F^{l}$:

(9) $$F^{h}=U\left(U\left(Conv\left(F^{\prime }\right)\right)\right),$$

(10) $$F^{l}=Conv\left(Conv\left(I\right)\right).$$

The high-level feature $F^{h}$ integrates both the image embedding from the encoder and the output embedding from the decoder; hence, it aggregates the semantic information of the input image. On the other hand, the low-level feature $F^{l}$ directly extracts features from the high-resolution image, and it preserves the detailed information of the furniture. In this way, we concatenate them and refine the segmentation map as

(11) $$S_{r}=Conv\left(Conv\left(Cat\left(F^{l},F^{h}\right)\right)\right).$$

The refined segmentation map $S_{r}$ is considered the final prediction; it is refined by the detailed feature from the original image, and it better preserves the structure of the furniture.

### 4.5. Network Training

In this work, the parameters of the original SAM encoder and mask decoder are frozen. We only update the parameters with a flame symbol, as shown in Figure 2. The proposed network produces three segmentation maps: (1) the coarse map $S_{c}$ by upsampling the image embedding *F*, (2) the output of the mask decoder $S_{o}$, and (3) the refined map $S_{r}$ via the SRM. The refined map $S_{r}$ is the final output, the coarse map $S_{c}$ and the output of the mask decoder $S_{o}$ are intermediate results, and both of these are supervised by the ground truth (GT). To train the proposed network, we introduce binary cross entropy (BCE) loss to the three segmentation maps:

(12) $$L=\alpha L_{\mathrm{BCE}}\left(S_{c},GT\right)+\beta L_{\mathrm{BCE}}\left(S_{o},GT\right)+\gamma L_{\mathrm{BCE}}\left(S_{r},GT\right),$$

where $L_{\mathrm{BCE}}=-\frac{1}{N}\sum_{i=1}^{N}\left[y_{i}\log\left({\hat{y}}_{i}\right)+\left(1-y_{i}\right)\log\left(1-{\hat{y}}_{i}\right)\right]$, *y* is the ground truth label (either 0 or 1), and $\hat{y}$ is the predicted probability. The coefficients α, β, and γ are the weights of the loss function. In the experiments, the coefficients α, β, and γ are all set to 1. The network is trained in an end-to-end manner with the loss function.

## 5. Results

### 5.1. Implementation Details

For a fair comparison, all models were retrained using the training set of MF2K with an input image resolution of 352×352. The training was stopped after 50 epochs, and the weights from the last epoch were used for all models. For our model, the batch size was set to 16, and the learning rate was initialized at 0.0001 and adaptively decreased during training using the Adam optimizer. Horizontal flipping and random cropping were applied for data augmentation. The training and testing were conducted on an NVIDIA 3090 GPU.

In the experiment, we conducted comparative experiments with U-Net [29], CPD [22], F3Net [23], GateNet [15], GCPANet [24], DASNet [21], ICON [14], and MDSAM [25]. For the first seven models, the parameters were fully tuned by the optimizer. For MDSAM [25] and our model, most parameters were frozen, and the partial parameters were tuned in a parameter-efficient fine-tuned manner.

### 5.2. Evaluation Metrics

In our evaluation, we adopted the mean intersection over union (mIoU) as the primary metric to measure the segmentation accuracy. The mIoU is a robust and widely used metric that quantifies the overlap between the predicted and ground truth masks. For each class, the intersection over union (IoU) is defined as

(13) $$\mathrm{mIoU}=\frac{1}{C}\sum_{i=1}^{C}\frac{|P_{i}\cap G_{i}|}{|P_{i}\cup G_{i}|},$$

where $P_{i}$ is the set of predicted pixels for class *i*, $G_{i}$ is the set of ground truth pixels for class *i*, $|P_{i}\cap G_{i}|$ is the number of pixels in the intersection of the predicted and ground truth sets, and $|P_{i}\cup G_{i}|$ is the number of pixels in the union of the predicted and ground truth sets. *C* is the total number of classes; in binary or single-class segmentation tasks, the mIoU evaluates the foreground versus background, whereas in multi-class segmentation, it accounts for each class’s segmentation quality. A higher mIoU value indicates better alignment between the predicted masks and the ground truth, reflecting the model’s ability to precisely delineate object boundaries and handle fine details. Given the intricate designs of Ming-style furniture in our MF2K dataset, the mIoU serves as an appropriate metric to evaluate how well each model captures the complex structure of the furniture.

### 5.3. Quantitative Comparisons

Table 2 presents a comparison of various models based on their backbone, tuned parameters, frames per second (FPS), and mIoU scores. The models in this comparison span a variety of backbones, including traditional convolutional networks such as ResNet50 and more recent transformer-based architectures such as ViT-B and Swin-B. The analysis focuses on the tuned parameters, computational efficiency (FPS), and segmentation accuracy (mIoU).

The baseline U-Net, which does not use a pretrained vision encoder, has a parameter count of 31 million and achieves an mIoU of 0.7968, with an FPS of 82. Although U-Net is relatively lightweight, it is outperformed in both speed and segmentation accuracy by more modern models.

Among the convolutional models, F3Net demonstrates a balanced performance with 26 million tuned parameters and the highest FPS (158), along with an mIoU of 0.8657. This makes F3Net the most efficient model in terms of speed while maintaining strong segmentation performance. CPD and GateNet also show competitive results, with CPD achieving an mIoU of 0.8585 and GateNet close behind at 0.8551, although GateNet has a much higher parameter count (128 million) than the other ResNet50-based models.

Transformer-based models such as ICON (Swin-B) and MDSAM (ViT-B) show a trade-off between the parameter efficiency and segmentation performance. ICON, with 94 million tuned parameters, achieves an mIoU of 0.8543, although its FPS is relatively low at 65. On the other hand, MDSAM has fewer parameters (11 million) and outperforms ICON with an mIoU of 0.8919, albeit at a slightly lower FPS of 50.

The proposed model stands out with the fewest tuned parameters (7 million) while achieving an mIoU of 0.9048, the highest among all compared models. Additionally, it maintains a competitive FPS (52), making it both efficient and highly accurate. Compared to MDSAM, which also uses the ViT-B backbone, our model achieves better segmentation performance with fewer parameters and a slightly higher FPS.

In summary, the proposed model achieves the best balance of accuracy and parameter efficiency, with the highest mIoU score of 0.9048 and the fewest tuned parameters (7 million). Its FPS of 52, though not the highest, is comparable to other high-performing models, making it an optimal choice for scenarios requiring both accuracy and computational efficiency. This makes our method highly suitable for tasks requiring high precision, such as the detailed segmentation of Ming-style furniture, while maintaining computational efficiency.

### 5.4. Qualitative Comparisons

**Figure 3:**
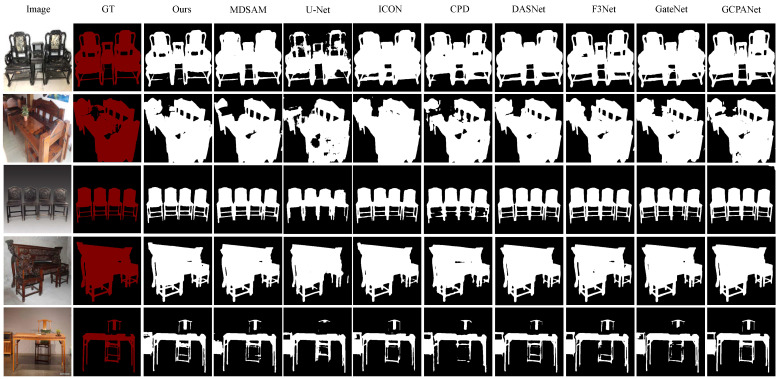
Visual comparison with state-of-the-art models. Our model demonstrates notable improvements in capturing intricate furniture boundaries and preserving structural integrity in the segmentation of Ming-style furniture images.

As shown in Figure 3, the qualitative comparison highlights our model’s ability to achieve finer segmentation accuracy, making it especially well suited for applications where high-resolution segmentation of detailed objects like Ming-style furniture is required.

For example, in the first and second rows, our model excels in capturing boundaries and structure in the segmentation of Ming-style furniture images, particularly evident in interior regions on the chair back. It consistently preserves fine interior patterns and accurately adheres to furniture boundaries, producing higher-quality segmentations compared to other models. Competing models often produce blurred boundaries around complex areas and miss interior structures. Based on the third row, the segmentation of our model preserves finer slender structures such as a chair leg, while competing models like MDSAM [25] and CPD [22] produce adhesive masks of a chair leg. Additionally, the fifth row shows that our model demonstrates robustness in distinguishing between furniture and challenging elements such as shadows or occlusions, which other models frequently misinterpret as part of the furniture.

### 5.5. Ablation Studies

The ablation study in Table 3 demonstrates the impact of the two proposed modules: the material attribute prompter (MAP) and the structure refinement module (SRM), both individually and combined. The first row shows the performance of the baseline with only adapters in the SAM encoder, and the mIoU is 0.8142. When the MAP is used, the model achieves the highest FPS (71) and a solid mIoU score of 0.8617, indicating that the MAP contributes significantly to the speed, while maintaining good segmentation accuracy. On the other hand, using only the SRM results in a lower FPS (66) and a slightly reduced accuracy (0.8492), showing that the SRM primarily enhances the segmentation quality at the cost of some computational speed.

When both modules are combined, the model achieves the best mIoU of 0.9048, reflecting the complementary strengths of the MAP and SRM in improving the segmentation performance. However, the FPS decreases to 52, indicating that the additional complexity introduced by both modules reduces the speed but provides a substantial boost in the segmentation accuracy. This confirms the effectiveness of combining the MAP and SRM to achieve the best balance between accuracy and efficiency.

The ablation study in Table 4 systematically evaluates the contributions of different components within the MAP. Equipped with only the SRM, the baseline mIoU is 0.8492. Adding the basic prompt (BP) improves the mIoU to 0.8640, demonstrating that the BP effectively enhances the segmentation. Introducing the contextual prompt (CP) further boosts the mIoU to 0.8772, indicating that the combination of the BP and CP significantly strengthens the performance.

When the coarse map $S_{c}$ supervision is incorporated, the mIoU increases to 0.8821, showing that the $S_{c}$ contributes to refining the mask prediction. Adding the attribute-guided attention mechanism $A_{\phi -F}$ enhances the mIoU to 0.8945, reflecting its importance in improving the model precision. Finally, with both the cross attention $A_{\phi -F}$ and $A_{F-\phi -F}$ included, the model achieves the highest mIoU of 0.9048, validating the complementary effects of these components in improving the segmentation quality. This progression highlights the effectiveness of each element in the MAP, showing how their integration leads to substantial improvements in the mIoU.

The ablation study within the structure refinement module (SRM) presented in Table 5 illustrates the contribution of different feature components to the overall segmentation performance, measured via the mIoU. The first row shows the performance of the baseline with only the MAP, where the mIoU achieves 0.8617. Adding $F^{d}$ alone increases the mIoU to 0.8733, which highlights the importance of leveraging features from the decoder. When both the $F^{d}$ and $F^{e}$ are included, the mIoU further improves to 0.8894, demonstrating that enhancing the representation by combining upsampled features from the encoder yields better results. Finally, incorporating all three components, including the $F^{l}$, achieves the highest mIoU of 0.9048, confirming the effectiveness of utilizing both high- and low-level feature refinements within the SRM.

## 6. Discussion and Applications

### 6.1. Discussion

As time passed, there were various influences between society, politics, economy, culture, ethnicity, and lifestyle, which led the development of Ming-style furniture to shift from prosperity to decline. With increasingly limited application, the transmission of this intangible cultural heritage is less likely. The concept of intangible cultural heritage began to spread around the world in the early 21st century, with various protective measures being implemented to raise awareness of the importance of living heritage. The application of artificial intelligence technology in cultural heritage protection and dissemination has evolved from initial information storage and transmission to data processing and analysis, and even further to today’s automated and intelligent processing and display. It has played a crucial role in the protection, research, documentation, and design application of cultural heritage. In the process of building a digital Ming-style furniture dataset, integrated technologies combining 3D scanning and photogrammetry are often used to create 3D models of furniture to achieve the purpose of dataset construction. However, due to the hardwood characteristics and large size of Ming-style furniture, the transportation process is complex. Moreover, the assembly of Ming-style furniture is performed using mortise-and-tenon structures, which are difficult to restore once disassembled without the assistance of professionals. Our research is beneficial for the preservation of the design, patterns, craftsmanship, and structure of Ming-style furniture, greatly reducing the damage to the artifacts and preserving the integrity of the furniture. This provides a safer solution for subsequent protection and innovation work on Ming-style furniture.

This study demonstrates a significant advancement in image segmentation for complex cultural artifacts, particularly Ming-style furniture, which is known for its intricate structures and details. Compared to previous segmentation models, the proposed approach with the material attribute prompter (MAP) and structure refinement module (SRM) achieves a superior mIoU score, highlighting its effectiveness in preserving fine-grained details. These findings align with prior research that emphasizes the importance of both feature refinement and context-specific prompts in segmentation tasks, extending such insights to cultural heritage applications. From the perspective of previous studies, the combination of the MAP and SRM confirms the hypothesis that material-specific prompts and multi-level feature refinement can address the limitations of generic models in segmenting complex high-detail objects. Unlike traditional segmentation approaches, which often struggle with high-frequency details in cultural artifacts, through leveraging both basic and contextual prompts along with high- and low-level feature integration, our model provides a tailored and efficient solution.

### 6.2. Applications

Furniture design and spatial layout are two indispensable aspects of modern architecture and interior design that influence each other to enhance user comfort and happiness. The research into the application of Ming-style furniture in modern space is relatively limited, which is not conducive to the transmission and development of intangible cultural heritage. This research could illustrate the design and matching issues of Ming-style furniture in different spaces. Identifying the matching patterns of Ming-style furniture in spatial design can demonstrate the compatibility of traditional intangible cultural heritage with modern environments, helping designers and related practitioners to improve the efficiency of home environment design and expand the business market for Ming-style furniture. The article’s dataset includes Ming-style furniture made from eight types of wood available on the market and classifies the spaces into tea rooms, corners, courtyards, bedrooms, studies, dining rooms, living rooms, and entryways. Our model, as a segmentation tool, can quickly identify the design regulation in spatial arrangements.

**Figure 4:**
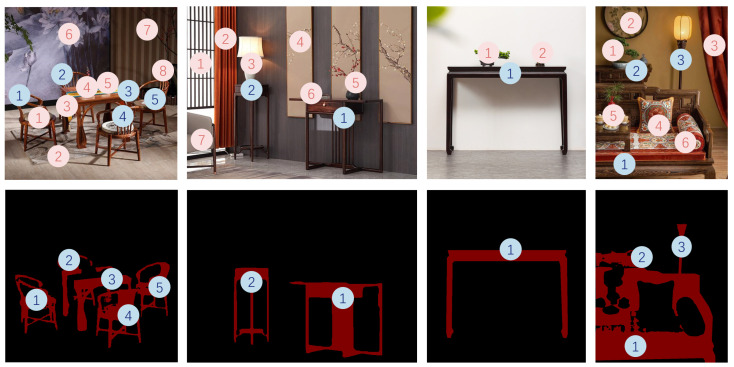
Quantitative relationship between Ming-style furniture and other decorations. The numbers in pink indicate Ming-style furniture, and the numbers in light blue indicate other decorations in the environment.

The quantitative relationship between Ming-style furniture and other decorations is shown in Figure 4. In the dining room, Ming-style furniture is often placed in the form of a furniture combination, paired with more than eight types of modern accessories to create a warm atmosphere. In the corner, 1–2 pieces of Ming-style furniture are collocated with more than seven modern decorative items to reflect a tranquil space. In bedroom spaces, Ming-style furniture, due to its robust material characteristics, is often matched with more soft furnishings to design a comfortable ambiance. In entryway scenarios, both furniture and decorations are reduced to reflect simplicity.

**Figure 5:**
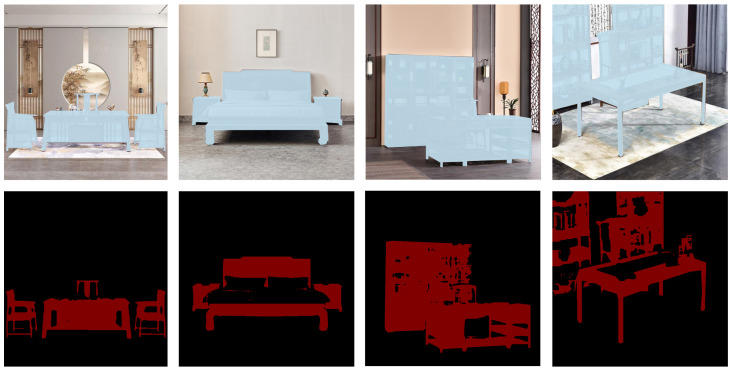
The spatial proportion relationship of Ming-style furniture. The red masks are generated from our segmentation model, and the pink masks are refined regions of the Ming-style furniture.

The spatial proportion relationship of Ming-style furniture is shown in Figure 5. The teahouse usually displays a furniture group composed of several pieces of Ming-style furniture, and it raises the ceiling distance, leaving the top space blank. In bedrooms, furniture ideally occupies one-third of the space to ensure comfort. In studies, extensive use of Ming-style furniture can enhance the professionalism and authority of the space.

## 7. Conclusions

This work presents a robust automated approach to segmenting complex objects in Ming-style furniture images, demonstrating clear improvements over previous models in both the segmentation accuracy and the preservation of fine details. By integrating the material attribute prompter (MAP) and the structure refinement module (SRM), the proposed model achieves superior performance, efficiently capturing intricate design elements that are critical for the preservation and digital documentation of cultural heritage. The combination of the MAP and SRM allows for significant advancements in refining the segmentation quality and addressing the challenges posed by complex structures and materials.

The key findings of this research highlight that the introduction of the MAP enables the automatic generation of material-specific prompts, significantly enhancing the segmentation precision without requiring extensive manual intervention. This reduces the dependency on domain expertise during the dataset annotation and accelerates the overall segmentation workflow. The SRM further complements this by integrating high-level semantic features with low-level details, ensuring that fine-grained structures, such as carvings and joints, are accurately preserved in the segmented outputs. These innovations collectively elevate the model’s ability to handle the unique challenges posed by the intricate designs of Ming-style furniture.

This study also contributes a newly constructed dataset, MF2K, which includes 2073 high-quality annotated images of Ming-style furniture, categorized across eight material types and various spatial environments. This dataset provides a valuable resource for future research, enabling the exploration of segmentation techniques in similarly complex and culturally significant domains. The dataset not only facilitates model training and evaluation but also serves as a benchmark for comparing segmentation approaches in cultural heritage applications.

The significance of this work lies in its potential to bridge the gap between advanced computer vision technologies and cultural heritage preservation. By providing a domain-specific solution tailored to the unique characteristics of Ming-style furniture, the research underscores the importance of contextualized technological innovation. This methodology aids conservationists in cataloging and analyzing historically significant pieces while reducing the physical handling of and potential risk to the objects. Moreover, the model’s efficient handling of the segmentation tasks fosters new opportunities for heritage research, including detailed pattern analysis, virtual restoration, and the creation of digital twins for education and outreach purposes.

Future research could extend this framework to other forms of cultural artifacts, adapt it to higher-resolution imagery for even more detailed segmentation, or explore its integration with emerging technologies such as augmented reality (AR) and virtual reality (VR). These applications could revolutionize the way cultural heritage is preserved, studied, and experienced, fostering greater global awareness and appreciation of intangible cultural assets. Furthermore, collaborations with museum professionals and historians could enable the creation of enriched datasets and contextual narratives, enhancing the broader impact of such technologies.

## Figures and Tables

**Table 1:** Data distribution of our dataset MF2K.

	Burmese Rosewood	Black Walnut	Siamese Rosewood	Yellow Rosewood	Chicken Wing Wood	Tielimu	Ceylon Ebony	Chinese Rosewood
Dining room	26	41	20	27	23	23	26	29
Tearoom	24	27	28	24	33	22	23	21
Corner	117	58	91	135	114	86	86	94
Living room	56	84	92	35	54	26	74	77
Study	24	23	29	32	36	22	26	23
Courtyard	51	20	33	30	24	34	19	26
Bedroom	25	33	57	23	27	19	43	27
Hall	36	40	28	37	34	22	35	35
Subtotal	359	326	378	343	345	254	332	332

**Table 2:** Comparison with state-of-the-art models in terms of the backbone, tuned parameters, FPS, and mIoU. The best performance of each metric is in bold text. The symbol “-” indicates that the network does not use a pretrained vision encoder. The symbol ↓ indicates that a lower value is better, and the symbol ↑ indicates that a larger value is better.

Method	Backbone	Tuned Parameters (M) ↓	FPS ↑	mIoU ↑
U-Net [29]	-	31	82	0.7968
CPD [22]	ResNet50	29	120	0.8585
F3Net [23]	ResNet50	26	**158**	0.8657
GateNet [24]	ResNet50	128	130	0.8551
GCPANet [24]	ResNet50	67	33	0.8646
DASNet [21]	ResNet50	37	18	0.8635
ICON [14]	Swin-B	94	65	0.8543
MDSAM [25]	ViT-B	11	50	0.8919
Ours	ViT-B	**7**	52	**0.9048**

**Table 3:** Ablation study of the proposed two main modules: the material attribute prompter (MAP) and the structure refinement module (SRM). The symbol ✓ means the component is adopted by the setting. The symbol ↓ indicates that a lower value is better, and the symbol ↑ indicates that a larger value is better.

MAP	SRM	FPS ↑	mIoU ↑
			0.8142
✓		**71**	0.8617
	✓	66	0.8492
✓	✓	52	**0.9048**

**Table 4:** Ablation study within the material attribute prompter (MAP). The symbol ✓ means the component is adopted by the setting, and the symbol ↑ indicates that a larger value is better.

BP	CP	$S_{c}$	$A_{\phi -F}$	$A_{F-\phi -F}$	mIoU ↑
					0.8492
✓					0.8640
✓	✓				0.8772
✓	✓	✓			0.8821
✓	✓	✓	✓		0.8945
✓	✓	✓	✓	✓	**0.9048**

**Table 5:** Ablation study within the structure refinement module (SRM). The symbol ✓ means the component is adopted by the setting, and the symbol ↑ indicates that a larger value is better.

$F^{d}$	$F^{e}$	$F^{l}$	mIoU ↑
			0.8617
✓			0.8733
✓	✓		0.8894
✓	✓	✓	**0.9048**

## Data Availability

The data presented in this study are available upon request from the corresponding author.
